# Building elder care training for migrants and refugees employed in informal care: suggestions from the SWOT analysis of the educational programme “HERO”

**DOI:** 10.3389/fpubh.2025.1628714

**Published:** 2025-09-24

**Authors:** Matteo Finco, Sara Santini, Sotiria Moza, Elena Kyprianou, Christina Yerou, Theologia Tsitsi, Maria Victoria Soulé, Andreas Charalambous, Panos Kassidakis, Julian Ulecia, Stavros Pitoglou, Flavia Galassi

**Affiliations:** ^1^Centre for Socio-Economic Research on Aging, IRCCS INRCA-National Institute of Health and Science on Aging, Ancona, Italy; ^2^Materia Group, Nicosia, Cyprus; ^3^Language Centre, Cyprus University of Technology, Limassol, Cyprus; ^4^Nursing Department, Cyprus University of Technology, Limassol, Cyprus; ^5^Department of French and European Studies, University of Cyprus, Nicosia, Cyprus; ^6^Department of Nursing, University of Turku, Turku, Finland; ^7^Aktios Elderly Care Units, Athens, Greece; ^8^SPSI, SA, Travessa da Praia 1, Lisbon, Portugal; ^9^Computer Solutions SA, Athens, Greece

**Keywords:** elder care, long-term care (LTC), migrants, refugees, health policy, training

## Abstract

**Background:**

The increased demand for care by older population with multimorbidity and the shortage of staff in the care sector are challenging healthcare systems across Europe. Migrants currently represent a valuable resource to bridge the gap between demand and supply of care in both the formal and informal elder care sector. Their specific educational and social needs have to be addressed by tailored training courses, which would allow them to provide quality care for older people at fair working conditions.

**Methods:**

The study analysed the perspective of 83 migrants and refugees participating in an elder care course implemented in 2021 in Cyprus, Greece, Italy and Portugal and 35 experts in adult education and elder care, who were involved in semi-structured interviews and focus groups, respectively. The textual content were analysed thematically, to identify strengths, weaknesses, opportunities and threats of the training.

**Results:**

Three main themes were identified that may characterise a successful and effective elder care training for migrants: useful educational content, lessons time flexibility and a meaningful relationship with the trainers. Results suggest the improvement of some educational aspects and the design of social investment policies that can recognise the acquired certification at the EU level, help trainees enter the labour market and older people have a good quality assistance.

**Discussion:**

Proper elder care can prevent the onset of very common risks for the health of older people with long-term care needs and then decrease the hospital accesses and the general pressure on the Health System. Policy recommendations are given framed in the social investment policy framework to consider and monitor all stages of the elder care supply chain, from education up to employment of migrant care workers.

## 1 Introduction

In many Western countries, the increasing demand for long-term care (LCT) by older people with multimorbidity (1) and a serious shortage of qualified staff in the formal care sector and of trained family caregivers in the informal one (2) are threatening the sustainability of the national health systems.

In the formal care sector (hospital and care facilities), some European national health systems (such as in Germany, Sweden, UK) are recruiting migrant care professionals to fill in the gap between the demand for long-term services and the offer of healthcare workforce (3, 4).

In the informal care sector, especially in the Mediterranean countries, characterised by familistic care regimes and less developed LTC services (5), informal caregivers tend to hire private care assistants, mostly migrants, to cover the lack of public home care and residential services (6). This is an attempt to guarantee person-centred and continuous care, that is, the possibility of ageing at home and delaying entry into residential care (7).

Migrant care workers (MCWs) therefore represent a fundamental human resource both in the formal and the informal elder care sector (8–12). Nevertheless, they may experience a number of precarious and unfair working conditions compared to local national colleagues both in the formal (13) and in the informal care sector (14, 15).

In most EU countries, having an elder care certification is not required for staff working providing care in people’s home as an indicator of capabilities (16). Nevertheless, the demand for elder care is so high that it is easy to find employment as a care assistant, even without certification, especially for those, who are willing to accept demanding shifts and low wages, like migrants (14, 15, 17).

In many European countries there are different vocational education and training (VET) programmes, career guidance services, continuous job training, and mentoring/coaching and psychological and social support interventions (18–21).

### 1.1 Refugees and migrants’ training offer in the study countries

Specific training in elder care for refugees and migrants are often unaccessible to most of the migrants and refugees because of high cost, the need to possess a High School Certificate degree and the requirement to have the B1 (medium) level of the Common European Framework of Reference for Languages (CEFR) (22).

Conversely, education can unlock the potential of MCWs (23). Healthcare training can have a great impact on employment prospects in the long-term, especially when updating is attended, and also in the short-term if the training lasts some weeks or months (24). VET programmes aimed to equip participants with practical skills and the know-how: they are considered to be a good start for further education, employment, and empowerment (25). To be effective, educational programmes for migrants have to ensure flexibility, pay attention to every single participant, give the possibility of training again in the future (24) and room to learners for expressing themselves, sharing experiences, and striving towards equality in the interaction (25). They are effective when they are learner-centred, based on problem solving abilities and linked to the needs of the labour market. However, they are more effective when they move beyond the simply idea of ‘imparting of knowledge’, and help people, especially young ones, develop tools and an attitude for continuous learning in the ever-changing world of work (26).

Ayalon and Shinan-Altman (27) underline the need for combining both a bottom-up and a top-down decision process when a training for MCWs is designed, in order to put together trainees, not-for profit organisations and employers’ vision and needs. In addition, engaging key parties in designing educational programmes for migrants and refugees, investing in trusted intermediaries, and addressing digital literacy and digital access, could be decisive for the success of the training (28).

Moreover, when migrants are involved, the training methodology should be interactive, taking into account different learning styles, providing an atmosphere of inclusion, and encouraging participation (29).

Language, communication, and cultural barriers are the main challenges in migrants’ education. In specific, the knowledge of the hosting country and the migrants’ educational level affect the learners’ employability, since the higher the number of assigned hours for language learning, the higher the labour force participation opportunities (30).

Moreover, some practical aspects should be carefully considered as possible threats for elder care training for migrants. For example, the distance the trainees have to cover to reach the training site and the transportation they use; and the possibility to combine the training with a job, given that migrants often use public transport, have no relatives, and sometimes have to renounce training in order to work (20).

At a European level, a common and homogeneous set of criteria for the assessment of appropriateness (i.e., to what extent the training addresses learners’ needs) and effectiveness (i.e., the impact of the training on trainees’ life) of the provided education is still missing. Conversely, such an evaluation may be useful for designing proper educational programmes that might ensure fairer working conditions for migrant care workers and higher standard of care for older people (25).

Moreover, noteworthy, evaluations of educational interventions at country level, highlight a potential gap between the idealised goals of training care workers to provide social and emotional support and the practical demands of older adults, who often require assistance with basic activities of daily living such as bathing, feeding, and mobility (27).

### 1.2 The study

The study is an evaluation of strengths, weaknesses, opportunities and threats (SWOT analysis) of the course developed within the project HERO-“*Training Program in Elderly Care and Infectious Disease Prevention for the Integration of Refugees from Middle Eastern and African Countries into Western Society*.” The course was implemented in 2022 in four countries – Cyprus, Greece, Italy, and Portugal – considered the key destinations for many immigrants in Southern Europe.

## 2 Methods

### 2.1 The HERO programme

**Figure 1:**
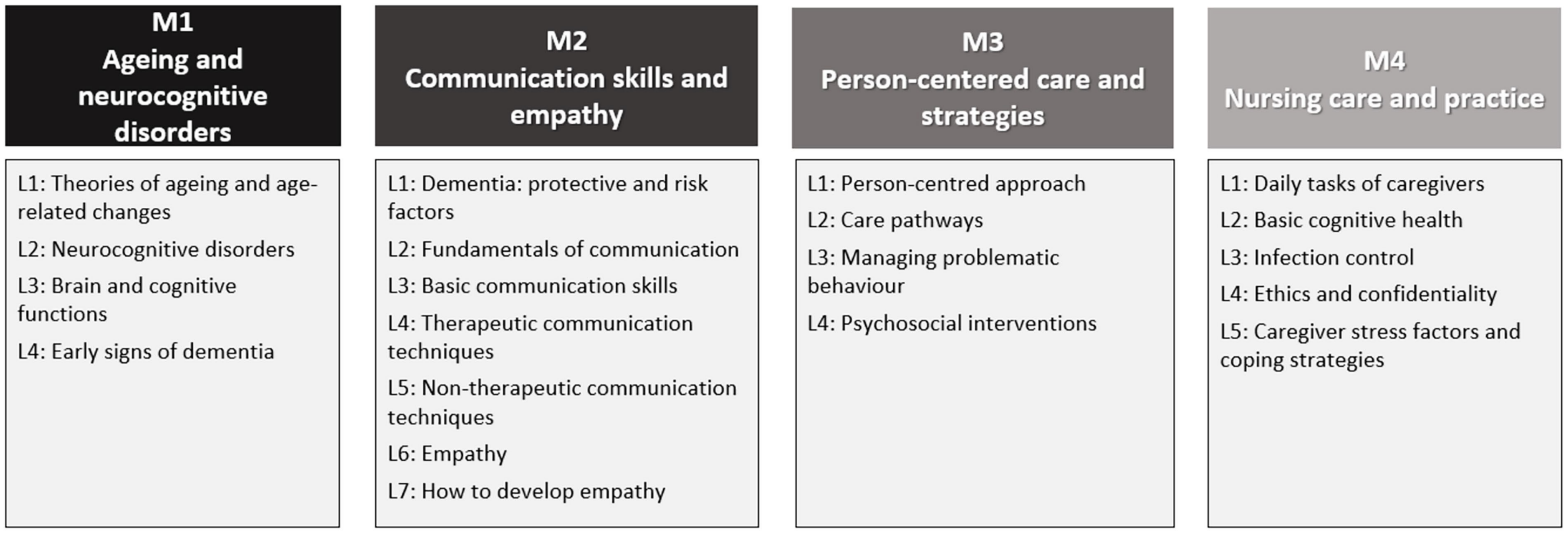
Training programme: modules (M) and lessons (L).

The training was carried out between June 2022 and October 2022 and its contents had been developed in English as well as in the three national languages, as described in (31) and summarised in Figure 1.

The overall 100-h training included 78 h of face-to-face lessons (24 h of language training and 54 h of elder care training), and 22 h of internship that was hosted by elder care facilities and geriatric hospitals/units. The internship was mainly practical, excluding the trainees in Italy, where it was mostly limited to observation (and not practical) due to internal insurance issues within the geriatric hospital, and due to the presence of numerous cases of Covid-19 that required virus containment procedures. In addition, an e-learning platform, following the format of a Massive Open Online Course (MOOC), was developed in order to provide online access to the course materials to all interested learners (link to the MOOC).^1^

### 2.2 Study design

**Figure 2:**
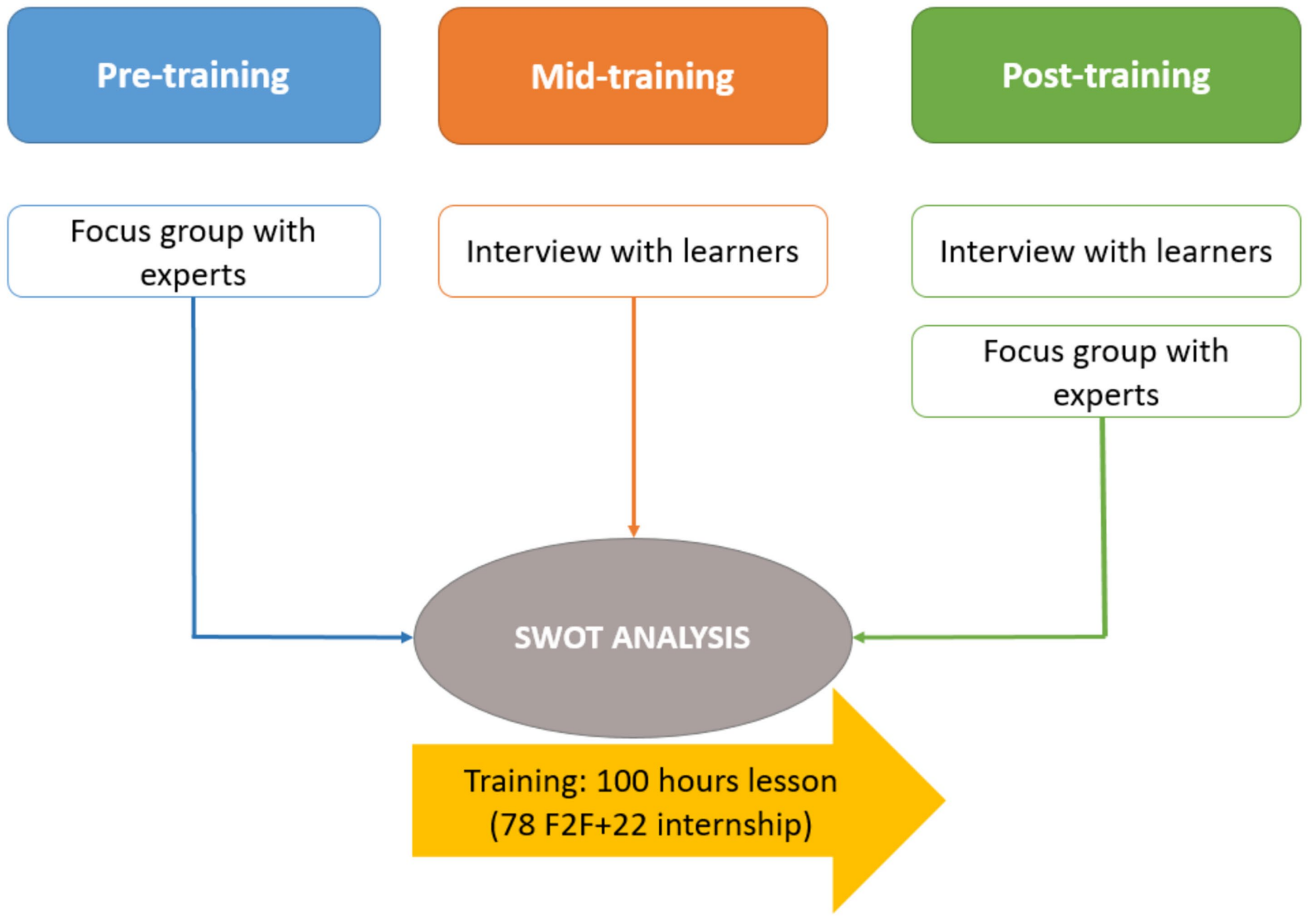
SWOT analysis design.

The SWOT analysis was realised based on the experience and knowledge of both learners (i.e., migrants and refugees) and professionals in adult education, elder care and migration, i.e., care unit managers, nurses, teachers/educators (Figure 2). The four key elements of the SWOT analysis are identified as follows. Strengths” (S) refers to the advantageous or positive characteristics of the educational programme enabling it to achieve its objectives (i.e., improving trainees’ quality of life, social inclusion and employment). “Weaknesses” (W) refers the negative or disadvantageous characteristics within the programme, representing obstacles to the desired objectives. “Opportunities” (O) denotes the elements in the external environment of the HERO programme that could be exploited to improve its efficacy. “Threats” (T) refers to the exogenous factors that could prevent the programme from achieving its goals. Gaining insight into these elements indeed, may provide knowledge about how educational programmes similar to HERO operate, so that improving actions can be undertaken strategically and systematically (32, 33).

### 2.3 Participants’ recruitment criteria

Migrants and refugees were recruited through NGOs. To be included in the study (i.e., in the training programme and in the evaluation), they had to have: (a) knowledge of the host country language corresponding to the A1 level (the basic one) of the CEFR for Languages; (b) a work permit or the status of refugee; (c) signed the informed consent to take part in the study. Moreover, at least 50 percent of participants had to be from African and Middle Eastern countries to comply with the original project idea.

Experts were mostly recruited within the study healthcare organisation network. No stringent inclusion criteria were applied for them. They were included in the study on the basis of their experience in the elder care sector or in the field of migration, vocational education, health and social services, such that they could bring different knowledge and experience, from frontline services to volunteering and policy in multiple sectors (i.e., elder care, migration, education) and could have direct experience of the educational programme for evaluating it properly. To achieve this goal, the majority of participants – alongside policymakers and education experts – were care unit managers, care professionals, and nurses who trained and tutored migrants during their internships. The topics addressed in the discussion were agreed by the different partners.

### 2.4 Characteristics of participants

In total, 83 trainees and 35 professionals were involved in the four study countries (Table 1): 20 trainees attended the course in Cyprus, 24 in Greece, 12 in Italy, and 27 in Portugal; 74.4% of the trainees were female whose average age was 33 years old (Table 2).

**Table 1:** Characteristics of trainees.

	Cyprus	Greece	Italy	Portugal	Total
Trainees, *n* (%)	20 (24.1)	24 (28.9)	12 (14.5)	27 (32.5)	83 (100)
Sex					
Male	8 (9.6)	8 (9.6)	4 (4.8)	1 (1.2)	21 (25.3)
Female	12 (14.5)	16 (19.3)	8 (9.6)	26 (31.3)	62 (74.7)
Age, mean	30.1	34.2	31.2	34.6	33.0
Place of birth					
Middle-East	0 (0.0)	7 (8.4)	2 (2.4)	0 (0.0)	9 (10.8)
Asia	5 (6.0)	1 (1.2)	1 (1.2)	0 (0.0)	7 (8.4)
North Africa	0 (0.0)	0 (0.0)	2 (2.4)	0 (0.0)	2 (2.4)
Central-Africa	15 (18.1)	16 (19.3)	6 (7.2)	7 (8.4)	44 (48.9)
South America	0 (0.0)	0 (0.0)	1 (1.2)	19 (22.9)	20 (24.1)
Europe	0 (0.0)	0 (0.0)	0 (0.0)	1 (1.2)	1 (1.2)
Highest education					
Early childhood	0 (0.0)	0 (0.0)	1 (1.2)	0 (0.0)	1 (1.2)
Primary	1 (1.2)	2 (2.4)	2 (2.4)	3 (3.6)	8 (9.6)
Lower secondary	0 (0.0)	3 (50.0)	3 (3.6)	0 (0.0)	6 (7.2)
Upper secondary	6 (7.2)	1 (1.2)	3 (3.6)	14 (16.9)	24 (28.9)
Post-secondary non-tertiary	1 (1.2)	3 (3.6%)	0 (0.0)	0 (0.0)	4 (4.8)
Short-cycle tertiary	0 (0.0)	7 (8.4)	1 (1.2)	0 (0.0)	8 (9.6)
Bachelor’s or equivalent	10 (12.0)	8 (27.6)	2 (4.5)	9 (10.8)	29 (34.9)
Master’s or equivalent	1 (1.2)	0 (0.0)	0 (0.0)	1 (1.2)	2 (2.4)
Previous certification in healthcare	10 (29.4)	9 (10.8)	4 (4.8)	11 (13.3)	34 (41.0)

**Table 2:** Characteristics of experts.

Experts, *n* (%)	9 (34.6)	10 (28.6)	12 (34.3)	4 (11.4)	35 (100.0)
Sex					
Males	0 (0.0)	4 (11.4)	4 (11.4)	0 (0.0)	8 (22.9)
Females	9 (25.7)	6 (17.1)	8 (22.9)	4 (11.4)	27 (77.1)
Profession					
Care managers/professionals	7 (20.0)	8 (22.9)	9 (25.7)	2 (5.7)	26 (74.3)
Policy makers	1 (2.9)	1 (2.9)	1 (2.9)	1 (2.9)	4 (11.4)
Experts in adult education	1 (2.9)	1 (2.9)	2 (5.7)	1 (2.9)	5 (14.3)
Total, *n* (%)	9 (25.7)	10 (28.6)	12 (34.3)	4 (11.4)	35 (100.0)

The majority of trainees in Cyprus, Greece and Italy were born in Central Africa (15, 16 and 6 respectively). In Portugal, however, the majority (i.e., 19 out of 27) were born in South America and migrated to Portugal due to the familiarity with the local language.

Considering all countries involved in the study, more than one third of the trainees had a higher educational degree (Bachelor’s or equivalent; only two had a master’s degree) with the highest percentage in Greece. 35 out of 83 trainees (41%) had already attended some kind of course in healthcare before HERO, receiving a certificate. However, it is worth mentioning that that the initial number of migrants and refugees registered in the training was higher. In fact, 21 of them answered the pre-training questionnaire and never attended the training, whilst 17 individuals dropped out from it. More details on drop-outs are provided in (31).

Out of 35 professionals, 27 were female. The most represented professions were care unit managers (CUM) and care professionals (CP) (74.3%), followed by experts in education (14.3%) and policy makers (13.4%).

### 2.5 Data collection

Migrants and refugees were interviewed halfway and after the training (see additional material for more details).

The mid-term interview detected any problem, concern, or difficulty that migrants and refugees were experiencing with the training and to eventually adjust content, plan, methods, and structure. Questions were: (a) Are you facing any difficulty during the training (for example with the topics, with patients or in the relationships with trainers and colleagues)? (b) What would you like to change? (c) Which are the positive aspects of the training?

The post-training interview asked strengths, weaknesses, opportunities, and threats of the programme according to the following topic-guide: (a) Which were the strengths of the training and what did you like most? (b) Can you tell me three threats/weaknesses of the training? (c) What did you dislike most? (d) Which were the main difficulties you had to face during the training? (e) In light of your answers above, what would you like to change in the training?

Professionals were involved in two focus groups: one before and another after the training delivery. In both waves, the discussion was led by four main questions: (a) Which are the HERO strengths? (b) Which are the HERO weaknesses? (c) Which are the HERO opportunities? (d) Which are the HERO threats?

In addition, in the post-training feedback, professionals were asked to answer the following questions on replicability and sustainability of the programme: (a) Which is the ‘lesson learned’ by the HERO experience? (b) Which is the suggestion you would like to give to the National/Regional health managers and policy makers about the training and the employment of migrants and refugees in the elder care sector in the country? (c) How can the training of migrants and refugees be funded and replicated in the future?

### 2.6 Data analysis

Textual data arising from interviews with migrants and refugees and focus groups with professionals were recorded and the content were transcribed verbatim in the national languages and only quotations addressing the study aims and answering the research questions were translated into English for cross-national comparison. The chunks of text were coded through a process that started deductively from the interview guide questions and continued inductively based on the answers of the interviewees. Then codes were grouped in themes that were thematically analysed (34) by the second author of this study with the support of MaxQda Plus software. The thematic analysis identified the main themes at the country level. The second step consisted in the comparison of themes by countries according to the Framework analysis (35) and in the perspective of the key elements of the SWOT analysis. The analysis and the classification of themes were first shared with national research themes and then discussed, revised, and refined by the first and the second author together, and again with the national research teams.

## 3 Findings

### 3.1 Learners’ perspective

#### 3.1.1 Mid-term feedback

Many trainees experienced difficulties in understanding the language, especially in relation to the more technical terms. Nevertheless, they stated that every aspect of the training was helpful, especially the internship, which allowed them to gain a better understanding and insight into the actual caregiving practise. A common suggestion was the use of videos during face-to-face lessons, because they found them more appealing and also because the images could help them understand the content, especially when their host country language *listening skills are* not very good.

Considering country specificities, in Cyprus, where the lessons were delivered online due to Covid-19 restrictions still in force, some trainees expressed the desire to have had more face-to-face lessons. Alternatively, as far as the positive aspects of the programme are concerned, the trainees appreciated the fact that they were given the opportunity to expand their social network and improve their quality of life by means of education. In Greece, some of the participants also mentioned that they had trouble operating the HERO training platform, but this was mainly due to digital illiteracy. In Portugal, trainees saw the opportunity to participate in the training programme as a way to be offered better conditions once they start applying for jobs. A trainee from the Portuguese context suggested that it would be very useful if they received some psychological support as ‘a plus’ to facilitate the experience.

In light of the mid-term feedback, trainers started using more videos during face-to-face lessons and the content were summarised in learning maps at the end of every lesson. Moreover, workshops on communication were conducted in small groups to help the interaction. In Italy, meetings were organised with a family caregiver (who was caring for his wife with severe disability), and a private care assistant (of older people with disability) to bring to learners the point of view of two actors who commonly paly pivotal roles in the home care setting of older people with disability living in Mediterranean countries. In Cyprus and Greece, trainees received technical support regarding their access to the online lessons. Psychological support was not directly provided because it had not been included as an objective of the project. However, since many trainers in the four countries were psychologists themselves, they emotionally supported the trainees during the training and helped them both in learning and interacting with peers and trainers.

#### 3.1.2 Post-training feedback

Table 3 shows the themes emerged from the Framework analysis of the interviews with learners by country (i.e., the cases). Specific quotations are reported in the manuscript body text.

**Table 3:** Trainees: themes by country and SWOT key elements.

Country	Key elements			
	S	W	O	T
Cyprus	Training content Collaboration trainers/trainees Flexibility	Online course	Keeping in contact with different cultures	Language barriers
Greece	Training content Free of charge	Logistic	Providing an opportunity of work	Certification not recognised
Italy	Training content Collaboration trainers/trainees	Language	Observational internship	Previous education not recognised
Portugal	Training content Collaboration trainers/trainees	Too much theoretical	Cultural barriers	Certification not recognised

Trainees considered the following dimensions as main strengths of the training: the content, the fact that the training was for free, the flexibility of times, and the relationship with trainers/teachers and peers. Offering an opportunity for finding a job, meeting other people experiencing similar difficulties and finding a new way for their integration in the community, were considered the main opportunities provided by HERO, as depicted by the following quotation examples from each country.

*‘The three strengths of the training: first is acquiring new knowledge and new environment and second is the teachers that also give us encouragement. The last strength of the training was to get to know different cultures. I mean different countries that include the teachers and students’* (Cyprus, ID 1, female, 28 years old, from Somalia).

*‘The main strength of the course was learning the Greek Language, learning processes on how to take care of people’* (Greece, ID 3, female, 21 years old, from Iran).

*‘A strength is that I met other friends and I have improved my language skills, as well’* (Italy, ID 13, female, 34 years old, from Tunisia).

*‘Giving a bath, knowing the body age transformations, the importance of taking good care of ourselves before we get old’* (Portugal, ID 24, female, 24 years old, from Angola).

The main weaknesses of the training vary from country to country. In Cyprus, trainees referred to problems of connectivity and low motivation of someone to attend the course systematically: *‘A weakness was that we did it online: it would have been better attending face-to-face in a class’* (Cyprus, ID 3, male, 21 years old, form Burundi), and *‘A weak point was the lack of motivation of some trainees who left the training’* (Cyprus, ID 2, male, 35 years old, from Nepal).

In Greece, the main weakness was identified in the logistics that for someone did not meet their needs: *‘The distance and the times of lessons were the main weaknesses of the training for me’* (Greece, ID 2, female, 58 years old, from Iran).

In Italy, the main complaint was the language and the fact that the lessons were theoretical, thus difficult to be understood by some trainees: *‘The language problem: I wanted to practise more than theory’* and *‘We could not understand all the words, let us say […] There was a lot of complex information to understand’.* Another weakness was identified in the internship that was only observational due to the Covid-19 restrictions still in force in the Italian hospitals: *‘I was a doctor in Somalia: more practise would be better to understand what to do with older patients!’* (Italy, ID 5, male, 30 years old, from Somalia).

Moreover, in Italy there were four women with small children and for the first two lessons they encountered some difficulties in combining parental responsibilities and training. After talking about it with the training organisers, the latter asked a psychologist to look after the trainees’ children whilst they were attending the lessons: *‘At the beginning I had a problem with where to leave the children, then it was solved’* (Italy, ID 13, female, 34 years old, from Tunisia).

Similarly, in Portugal, participants said that some trainers were not very aware that some students had never been in contact with older patients and that the theory could cause confusion without any previous practise or experience: *‘Some trainers are not very aware that some students never had the experience, so the theoretical part can be confusing’* (Portugal, ID 27, female, 25 years old, from Brasil).

In both Italy and Greece trainees appreciated the opportunity of meeting young people who experienced similar difficulties and who had similar needs: *‘The course gave me the opportunity to get to know so many young people from other countries and being together, living with different people’* (Italy, ID 21, female, 50 years old, from Peru) and *‘I made fine relationships with the rest of the trainees. We shared some of our experiences’* (Greece, ID 1, male, 37 years old, from Guinea).

Among the migrants and refugees attending the training in Greece, the most evident opportunity of the course was the increased chances of finding a job: *‘The course provided me with the opportunity to become more active and useful to the community’* (Greece, ID 24, female, 33 years old, from Cameroon).

The main threats to the fruition of the course are: (a) the language barrier, which remained until the end of the course despite the fact that the first module was on the hosting country care-related language and terminology (mainly in Cyprus), (b) the lack of a European common certification of the acquired competences (mostly in Greece and Portugal); and (c) the long hard process required for the recognition of training in healthcare for those who were doctors or nurses in their country. This opinion particularly concerns the trainees who attended the course in Italy, where 3 participants out of 12 had medical or nursing degrees (i.e., one physician and two nurses), and the fact that they could not do the work for which they had studied was a reason for frustration. This is not something of secondary importance: in fact, in several European countries, immigrants regularly work as nurses and physicians (36).

*‘The training and especially the internship bothered me, because for 10 years I worked as a nurse in my country and here, when I took the blood pressure of an old person, a nurse came and scolded me because it was not my job. That made me feel bad because I know the nursing work!’* (Italy, ID 8, 33 years old, from Afghanistan).

Trainees suggested improving the e-learning platform to make it more user-friendly, having face-to-face lessons instead of online ones and receiving support for finding a job as part of the course.

#### 3.1.3 Professionals’ training feedback

**Figure 3:**
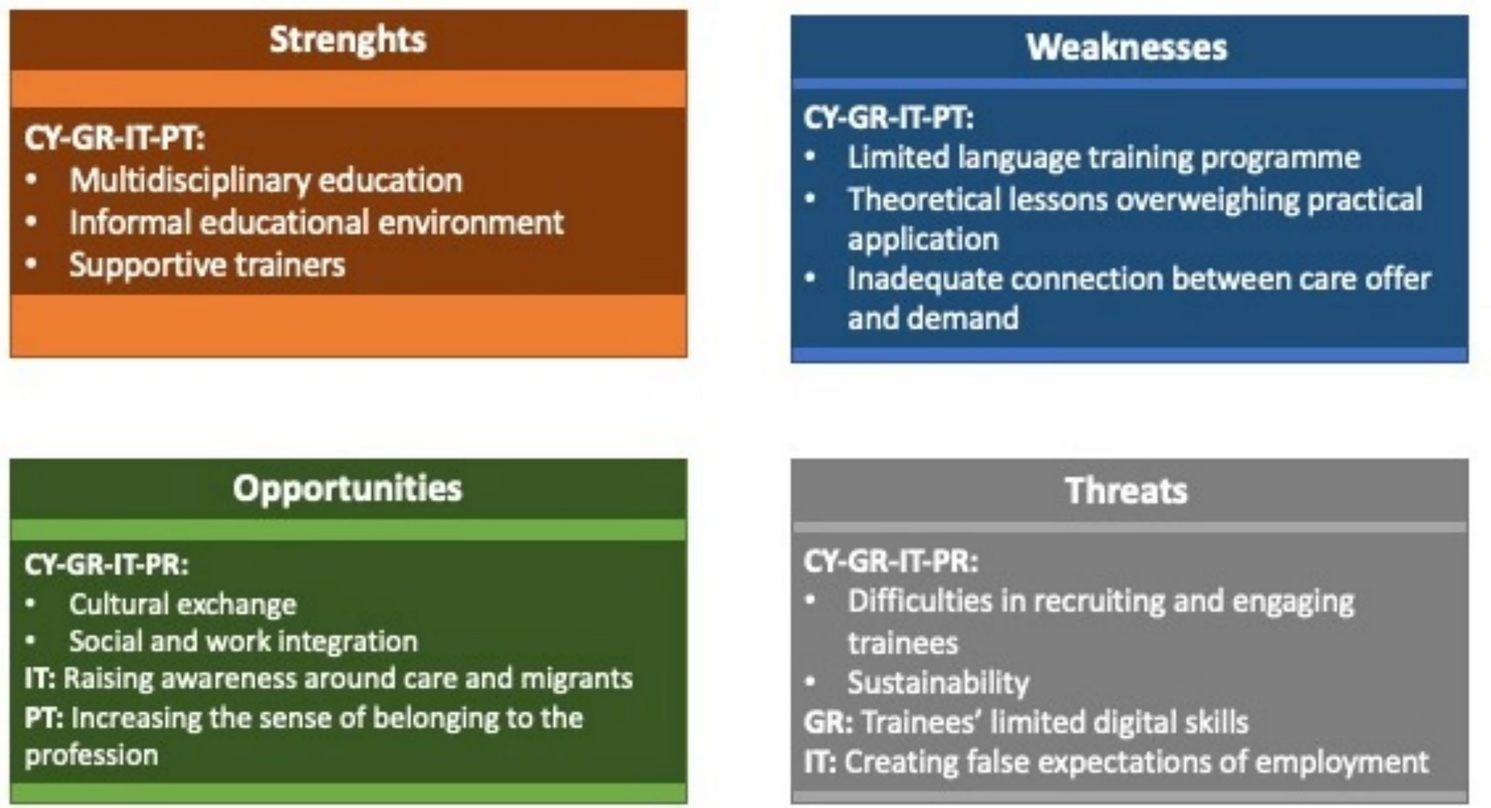
Experts: themes by country and SWOT key elements.

Cross-country common and country specific themes arisen from the post-training focus group, are reported in Figure 3 and are deepened in the text by means of the most meaningful quotations.

Experts in the four countries recognised the multidisciplinary of the educational content as a strength of the curriculum, which they found informative, rich, and useful: *‘Multidisciplinary content. I think this is very different from other training programmes in this area’* (EE, Portugal); and *‘I’m very surprised at the approach and then the initial, let us say, connection between the need and the resource, so it’s something that I think is completely new, at least for me’* (PM, Italy).

In Greece and in Portugal, the informal educational environment helped to create a trustful relationship between trainees and trainers. This enhanced the learning process, the recognition of and the identification with the hosting organisation’s mission and vision: *‘All the trainees were happy to have met us [trainers and tutors] and our care unit and they reported that they were impressed by the vision of the care unit and the approach we use to treat older patients’* (CP, Greece).

Three main weaknesses were observed in every country. Firstly, the length of the module that focused on the hosting country language: it was considered too short to fill the serious language gaps of most trainees. Secondly, the curriculum contained too many theoretical lessons which resulted in being difficult for trainees to follow and understand. Thirdly, experts in education and policy makers, especially from Italy, underlined the risk that educational programmes like HERO may remain disconnected from the labour market and they agreed on the necessity that any educational training should be linked to a programme for the migrants’ work inclusion to increase their employability: *‘The argument is that this kind of preparation, of training, should be within a higher project of offering the workforce’* (PM, Italy).

In addition, Italian professionals recognised that the observational internship limited the trainees’ knowledge of many practical and interactional aspects of taking care of an older person (e.g., how to change a diaper, how to feed and dress, etc.). They also highlighted the need for an internship in the older people’s home rather than one in the hospital, since the trainees received the certification of ‘Family assistant’, which allows them to work in the home care sector but not in hospitals: *‘I think trainees need more than just internship in the hospital. There has been a lack of internship on the ground, and I hope it will be organised in the next editions of the course, because trainees will go to work as caregivers in the homes of older people and, there, they will be alone with patients’* (CP, Italy).

In Portugal, a care unit manager underlined that there were some cultural barriers preventing participants from the full understanding of the educational content. This required that *‘some content (*e.g.*, infection prevention) were reformulated in compliance with the reality and the rules in force in the Country’* (CUM, Portugal). The main opportunity of the programme that was identified by all national professionals was the integration of migrants and refugees in the hosting countries’ society. In fact, during the programme, trainees met other trainees from the same native country as well as from different countries, including European trainers. In Portugal, policy makers underlined the potential of the course as *‘sharing knowledge, increasing the sense of belonging to the profession, encouraging discussion, putting in perspective’* (PM, Portugal).

In Greece, care managers underlined the value of the training for helping migrants find a job: *‘I expect that the training may help trainees adjust quickly in the workplace’* (CUM, Greece).

In Italy also arose the opportunity that the programme was intended to raise awareness around migrants and refugees as a resource for the society and as a possible answer to the dearth of care workers, that is questioning the health systems in the Western societies.

Finally, cultural differences were conceptualised as a resource from the very beginning of the HERO training. They were not a source of bias but rather an opportunity for discussion, as illustrated by the following quotation: *‘The only situation in which the cultural aspect represented a bias was with regard to circumcision and its relationship with infections, a topic on which a small debate was opened with an intern of African origin for whom the lack of circumcision of the majority part of our men was the reason for all urinary tract infections in older males’* (CP, Italy).

As far as the threats are concerned, the coordinator of an Italian course of nursing science expressed his concerns about the false expectations the course might transfer to the participants: *‘The primary weakness of the training lies in the trainees’ expectations of immediate employment and the limited practicality of the “family assistant” title’* (EE, Italy). In Greece, care managers and trainers thought that another threat for future courses may be *‘the limited knowledge and access to technology by migrants’* (CUM & EE, Greece) that may limit the chances of overcoming space and time constrains and having the opportunity of learning in any time.

A threat common to all four study sites was identified by the experts: the difficulty in recruiting and engaging migrants, mainly due to their need for work and the language barriers, that discouraged migrants to attend training programmes. In turn, the lack of language competences among the trainees blocked learning and participation. Another weakness was the strong element of theory in the lessons: ‘*In my opinion, the lessons were too theoretical, and the language level of the trainees was too poor for understanding such a* var*iety of content’* (Nursing trainer, Italy).

The economic sustainability of the training was identified as a possible threat by the experts in the four countries, who mentioned that the pilot results should drive policy makers and local authorities in the healthcare and educational sector to take the decision of funding other training editions: *‘Greater investment in training and that it is seen as something continuous. So people feel this is something important for their own development’* (CP, Portugal). The main suggestions for improving the training sustainability and replicability were: enhancing further project development (Cyprus); providing a certification that is valid at country as well as at European level that was appealing and increased the demand for elder care courses for migrants (Greece); building a network including research, care unit, regional authority, and NGOs (Italy); foreseeing longer and paid internship promoted/offered by the Government (Portugal).

In Italy, an expert in adult education underlined the possibility of asking for a small payment (a sort of co-participation to the expenses) for trainees, in order to motivate them to attend, and she also suggested to adopt a strategy for supporting the employment of migrants and refugees who achieved the educational certification.

## 4 Discussion

This study analysed strengths, weaknesses, opportunities and threats of a course aimed at training migrants and refugees on how to care for older people with long-term care needs in their own houses. This represents the main study novelty, given that most elder care courses train people on working in the formal care sector, i.e., in elder facilities and hospitals.

The study confirms that the cost for attending an elder care training may represent the first barrier to education and a source of inequality for migrants and refugees, because they may experience economic and social deprivation and they cannot afford to pay the costs of participation in the course (22).

Another barrier could be the language of the course: both learners and experts considered this aspect a weakness, since a limited comprehension of content by trainees represent a limitation for the social interaction (22). Moreover, even when the content is acquired, low language skill could limit the interaction and communication between the foreign caregivers and their patients.

In line with the literature (25, 26), the study confirms that educational programmes on elder care could facilitate the social inclusion of migrants and refugees, when there is a supportive relationship between trainers and trainees and an educational environment which helps inclusion, makes learners feeling accepted and enhances self-expression. In fact, in since many of the migrants and refugees attending the course had a background of poverty, uncertainty, broken family relationships, abuse and violence, they often come to the classroom with the primary need to feel safe and welcomed. Only when this need is met, can they actually learn. Moreover, the study underlined the importance of addressing the topic of cultural differences in the elder care practise and representations both in content (i.e., lessons topic) and in the class work (i.e., by working in multicultural groups) (29).

Contrary to what was suggested by Zeidman and Alaniz (28), the online mode was not appreciated by learners. This could due to the respondents’ low digital literacy and the need for socialisation that was mirrored by the willingness to interact with trainers and peers in the learning process.

The study also identified the need for less theoretical and more practical teaching approach to make training more appealing and interesting (26). Noteworthy, despite the majority if experts found the curriculum informative and exhaustive, some trainees found some lessons difficult to understand, too much theoretical and not appealing, and that therefore, they wanted more practical experiences. This underlines that trainees and experts had different viewpoints on the contents and teaching style and highlights the asymmetry between healthcare professionals and adult trainees in elder care with a migration background. This result again emphasises the importance of co-design so that these different perspectives are made to dialogue. From the perspective of implementation and management, the lack of a proper connection and cooperation among the public healthcare body implementing the training, the regional authority providing the certification of “family care assistant,” non-profit organisations, as possible trusted intermediaries and the public work office undermined the effectiveness of the training by reducing the employability of learners (28).

### 4.1 Training suggestions

**Figure 4:**
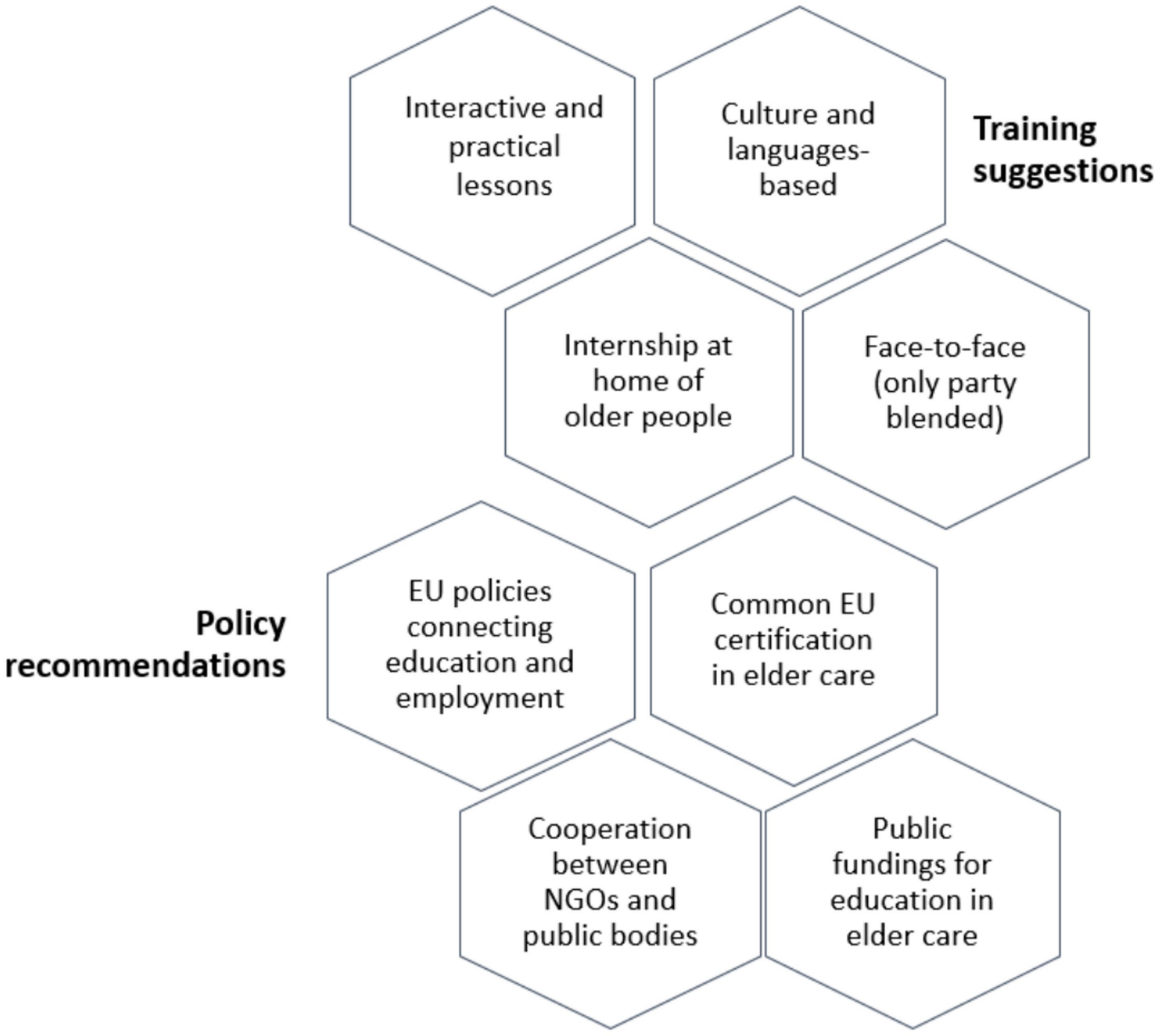
Training suggestions and policy recommendations.

Based on the findings, in this section and in the following one, several suggestions for future trainings and policy are given as shown in Figure 4.

First, elder care trainings for migrants should be constructed and conducted considering cultural and language differences, viewed as an opportunity for confrontation, and they should give learners room for expressing themselves and socialising. It is also recommended to select trainers who are not only trained on ageing process and healthcare, but that are also well-versed hospitality, psychological support, and are able to embrace diversity, such as for example ethnopsychologist and sociologists. Moreover, since the low level of linguistic knowledge may obstacle the learning process, the course should include a long linguistic module or, alternatively, the support of professional linguistic/cultural mediators should be taken into consideration, in order to help the trainees understand the content illustrated, together with the cultural and institutional context of the country, and, above all, establish dialogue across differences (37).

In addition, the trainees’ motivation to provide care and assistance for older people should be investigated before starting the training. Besides, attendance to such a training should not only be driven by the need and urgency to work, but also by a specific inclination and interest.

It would be important that training programmes include more care practise as suggested by the interviewed trainees (Table 3). A structured internship at home of the older persons in need of care, might ensure a higher quality of this kind of vocational education, and make learners more competitive in the informal care market. Nevertheless, an internship of 6 months, for instance, seems to be hard to foresee for at least two reasons. The first one concerns the socio-economic condition of migrants and refugees who may not reconcile internship with work and family. In fact, men have to make two or more temporary, low paid and often not regular jobs for addressing primary needs, whilst women have childcare responsibilities, no family network that may hinder the regular participation to the internship. Secondly, an internship in the domestic setting would require very strong cooperation between different agencies and institutions (such as educational organisations, police office, health units, etc.) and families of older persons, in order to guarantee the safety and privacy of both trainees and older patients. Also, specific tutors would be necessary, in order to follow the trainees in all course phases and coordinate them with healthcare personnel working in the facilities.

The study results calls for interactive lesson and more practical and less theoretical teaching approach.

For making the training more appealing and improving andragogical and didactical practise tailored around the needs of migrants.

Finally, trainings should be taken face-to-face for providing the chance for live interactions and human relationships and addressing the need of connection and interaction of the target. However, digital lessons may be a means for deepening certain topics and providing additional didactic materials, given that it can ease access to the content easier and promote flexibility, allowing those with practical and family responsibilities to be trained (20).

### 4.2 Policy recommendations

The results call for policies promoting the integration of informal education with formal one at national and/or regional level, according to the country legislation in the field of vocational education.

Integrated policies at a European level seem to be needed, and these should consider and monitor all stages of the care supply chain, from education of care workers, migrants or not, through the assessment of the knowledge and competencies up to employment. This is essential for both helping (migrant) caregivers to become employable, but also for families to find reliable, prepared and skilled home caregivers for their older relatives.

Moreover, as underlined by Ayalon and Shinan-Altman (27), policies are needed that get the educational system (training providers, schools, universities, etc.) connected to the labour market, and in cooperation with the associations working for the integration of migrants and refugees, such that their credentials as healthcare professionals acquired in their home country could be quickly recognised in the hosting country.

Furthermore, it is of crucial importance that the certification of the trainings developed through the European Commission funding programmes, such as HERO, could be recognised at a European level, particularly certifications addressing migrants and refugees, who often move from a country to another across Europe.

The policies described so far could be inscribed in the social investment policy framework (38), which pushes welfare systems to shift from a compensatory perspective (providing for a socio-economic need) to one of promoting individuals and groups. This paradigm envisages policies that: increase and maintain the stock of human capital and translate this into education and training programmes; facilitate the flow of gender transitions throughout life and into the labour market through measures to support work-life balance; and provide social buffers through economic support. According to the Migration Policy Institute Europe (39), social investment orientation can meet the needs of the 21st century society in which health, migration, work, family, and education are deeply intertwined. In this perspective, elder care training of migrants and refugees can contribute to increasing and maintaining the ‘stock’ of human capital by giving them the skills for long-term autonomy and employability – especially for women who usually have greater difficulties to enter the labour market. At the same time, this means high-quality standards healthcare and helps the reconciliation of work and care of informal caregivers. Having said this, to design elder care training for migrants and refugees and address their social and educational needs may represent the golden key for addressing the long-term sustainability of the health systems in an ageing society.

### 4.3 Limitations

Several weaknesses of the training might have limited this SWOT analysis as well. The most evident one is the language. In fact, the low language proficiency of many trainees might have affected their capability of expressing feedback about the course and their personal experience with it.

Moreover, it is possible that the considerations of the trainees on the educational experience itself might be influenced by the sense of gratitude towards teachers and researchers. Nevertheless, they felt free to make constructive critique, such as the lack of recognition of previous training, the linguistic difficulties of the course, and the fact that the internship was observational instead of practical. This was possible thanks to the strong cooperative relationship established between trainers and trainees in the non-formal adult education setting, in which trainees tried to minimise the social and educational asymmetry that, on the contrary, is common in the formal education.

Other limitations are embodied in the SWOT analysis. Although the analysis was not aimed at providing a gender/sex perspective (in fact the sex was not a criterion for the sample stratification), another limitation of the study is the lack of a gender analysis of the responses of both trainees and trainers, also due to the very limited number of individuals involved, who cannot be considered representative of the immigrant population. Since the care work as well as the educational work sector are strongly gender-driven, it may be useful to understand if male trainees, policy makers, care unit managers have a different perspective on which lessons, modalities, approaches are more useful than others, compared to females.

The second limitation relates to the impossibility to compare opinions by migrants’ nationality, because there was a high heterogeneity in terms of country of origin, languages and dialects in every national trainee group (Italian, Greek, and Portuguese) and a very limited number of subjects involved in the study.

## Data Availability

The original contributions presented in the study are included in the article/Supplementary material, further inquiries can be directed to the corresponding author.

## Footnotes

^1^ https://hero-platform.csl.gr//
