# Supplementary material for: Building elder care training for migrants and refugees employed in informal care: suggestions from the SWOT analysis of the educational programme “HERO”
Source: Front Public Health. 2025 Sep 24;13:1628714. doi: 10.3389/fpubh.2025.1628714 (PMC12504212; doi:10.3389/fpubh.2025.1628714)
Supplement: Supplementary file 1 [file Table_1.DOCX]

Supplementary Material

1. **PRE-PILOT QUESTIONNAIRE FOR THE TRAINEES (T0)**
2. **ABOUT YOU**

Before you begin we would like to ask you to answer a few general questions about yourself: by circling the correct answer or by filling in the space provided.

1. What is your gender?

| Male | 1 |
| --- | --- |
| Female | 2 |
|  |  |

1. **What is your date of birth?** ________ / ________ / ________
2. **How old are you?**_____________years
3. **Which is your birth country? Please, write it here: _____________________________**
4. **What is your marital status?**

| Single Separated | 0 |
| --- | --- |
| Married | 1 |
| Divorced | 2 |
| Living as married | 3 |
| Widowed | 4 |

1. **Have you got any child/ren?**

| No | 0 |
| --- | --- |
| Yes | 1 |

1. **How many children do you have?____________________**
2. **If you have any child/ren, how old is/are she/he/Them?  Please, write the age of your children here:**

| **Child** | **Age** |
| --- | --- |
| **1** |  |
| **2** |  |
| **3** |  |
| **4** |  |

1. **ABOUT YOUR EDUCATION**
2. **What is the highest education you received? Please, tick the number in the left column better representing your educational level**

| **Educational Level*** | **ISCED 2011*** | **Description*** | **Broad field of education****  Note for the interviewer: Please, specify the respondent’s broad field of education by adding the corresponding code** |
| --- | --- | --- | --- |
| 0 | Early childhood education | Education designed to support early development in preparation for participation in school and society. Programmes designed for children below the age of 3. |  |
| 1 | Primary education | Programmes typically designed to provide students with fundamental skills in reading, writing and mathematics and to establish a solid foundation for learning. |  |
| 2 | Lowe secondary education | First stage of secondary education building on primary education, typically with a more subject-oriented curriculum. |  |
| 3 | Upper secondary education | Second/final stage of secondary education preparing for tertiary education and/or providing skills relevant to employment. Usually with an increased range of subject options and streams. |  |
| 4 | Post secondary non-tertiary education | Programmes providing learning experiences that build on secondary education and prepare for labour market entry and/or tertiary education. The content is broader than secondary but not as complex as tertiary education. |  |
| 5 | Short-cycle tertiary education | Short first tertiary programmes that are typically practically-based, occupationally-specific and prepare for labour market entry. These programmes may also provide a pathway to other tertiary programmes. |  |
| 6 | Bachelor's or equivalent | Programmes designed to provide intermediate academic and/or professional knowledge, skills and competencies leading to a first tertiary degree or equivalent qualification. |  |
| 7 | Master's or equivalent | Programmes designed to provide advanced academic and/or professional knowledge, skills and competencies leading to a second tertiary degree or equivalent qualification. |  |
| 8 | Doctorate or equivalent | Programmes designed primarily to lead to an advanced research qualification, usually concluding with the submission and defense of a substantive dissertation of publishable quality based on original research. |  |

1. **Have you got any certification in healthcare?**

| No | 0 |
| --- | --- |
| Yes | 1 |

1. **Have you ever attended training in healthcare before HERO?**

| No | 0 |
| --- | --- |
| Yes | 1 |

1. **If yes, how many months did it last?** _____________________________________
2. **If yes, when did you attend the training? Please, write the year (and if you remember the months) when you attended the training (for example: January-March 2019):** ____________
3. **Who organised the training?** __________________________________________________________________________________________
4. **ABOUT YOUR HEALTH**
5. **Are you currently ill?**

| No | 0 |
| --- | --- |
| Yes | 1 |

1. **If something is wrong with your health what do you think it is?**___________________________
2. **Did you contact a doctor?**

| No | 0 |
| --- | --- |
| Yes | 1 |

1. **THE WHOQOL-BREF**

**Instructions for interviewers**

This assessment asks how you feel about your quality of life, health, or other areas of your life. Please answer all the questions. If you are unsure about which response to give to a question, please choose the one that appears most appropriate. This can often be your first response.

Please keep in mind your standards, hopes, pleasures and concerns. We ask that you think about your life **in the last two weeks**. For example, thinking about the last two weeks, a question might ask:

| **r.** |  | Not at all | Not much | Moderately | A great deal | Completely |
| --- | --- | --- | --- | --- | --- | --- |
| 0 | **Do you get the kind of support from others that you need?** | 1 | 2 | 3 | 4 | 5 |

I should circle the number that best fits how much support you got from others over the last two weeks. So you would circle the number 4 if you got a great deal of support from others. You would circle number 1 if you did not get any of the support that you needed from others in the last two weeks.

Please read each question, assess your feelings, and circle the number on the scale for each question that gives the best answer for you.

|  |  | Very poor | Poor | Neither poor  nor good | Good | Very good |
| --- | --- | --- | --- | --- | --- | --- |
| 1 | How would you rate your quality of life? | 1 | 2 | 3 | 4 | 5 |

|  |  | Very dissatisfied | Dissatisfied | Neither satisfied  nor dissatisfied | Satisfied | Very satisfied |
| --- | --- | --- | --- | --- | --- | --- |
| 2 | How satisfied are you with your health? | 1 | 2 | 3 | 4 | 5 |

The following questions ask about how much you have experienced certain things in the last two weeks.

|  |  | Not at all | A little | A moderate amount | Very much | An extreme amount |
| --- | --- | --- | --- | --- | --- | --- |
| 3 | To what extent do you feel that (physical) pain prevents you from doing what you need to do? | 1 | 2 | 3 | 4 | 5 |
| 4 | How much do you need any medical  treatment to function in your daily life? | 1 | 2 | 3 | 4 | 5 |
| 5 | How much do you enjoy life? | 1 | 2 | 3 | 4 | 5 |
| 6 | To what extent do you feel your life to be meaningful? | 1 | 2 | 3 | 4 | 5 |
| 7 | How well are you able to concentrate? | 1 | 2 | 3 | 4 | 5 |
| 8 | How safe do you feel in your daily life? | 1 | 2 | 3 | 4 | 5 |
| 9 | How healthy is your physical environment? | 1 | 2 | 3 | 4 | 5 |
| 10 | Do you have enough energy for everyday life? | 1 | 2 | 3 | 4 | 5 |
| 11 | Are you able to accept your bodily appearance? | 1 | 2 | 3 | 4 | 5 |
| 12 | Do you have enough money to meet your needs? | 1 | 2 | 3 | 4 | 5 |
| 13 | How available to you is the information that you need in your day-to-day life? | 1 | 2 | 3 | 4 | 5 |
| 14 | To what extent do you have the opportunity for leisure activities? | 1 | 2 | 3 | 4 | 5 |

|  |  | Very poor | Poor | Neither poor  nor good | Good | Very good |
| --- | --- | --- | --- | --- | --- | --- |
| 15 | How well are you able to get around? | 1 | 2 | 3 | 4 | 5 |

The following questions ask you to say how good or satisfied you have felt about various aspects of your life **over the last two weeks**.

|  |  | Very dissatisfied | Dissatisfied | Neither satisfied  nor dissatisfied | Satisfied | Very satisfied |
| --- | --- | --- | --- | --- | --- | --- |
| 16 | How satisfied are you with your sleep? | 1 | 2 | 3 | 4 | 5 |
| 17 | How satisfied are you with your ability to perform your daily living activities? | 1 | 2 | 3 | 4 | 5 |
| 18 | How satisfied are you with your capacity for work? | 1 | 2 | 3 | 4 | 5 |
| 19 | How satisfied are you with yourself? | 1 | 2 | 3 | 4 | 5 |
| 20 | How satisfied are you with your personal relationships? | 1 | 2 | 3 | 4 | 5 |
| 21 | How satisfied are you with your sex life? | 1 | 2 | 3 | 4 | 5 |
| 22 | How satisfied are you with the support you get from your friends? | 1 | 2 | 3 | 4 | 5 |
| 23 | How satisfied are you with the conditions of your living place? | 1 | 2 | 3 | 4 | 5 |
| 24 | How satisfied are you with your access to health services? | 1 | 2 | 3 | 4 | 5 |
| 25 | How satisfied are you with your transport? | 1 | 2 | 3 | 4 | 5 |

The following question refers to how often you have felt or experienced certain things in the last two weeks.

|  |  | Never | Seldom | Quite often | Very often | Always |
| --- | --- | --- | --- | --- | --- | --- |
| 26 | How often do you have negative feelings such as blue mood, despair, anxiety, depression? | 1 | 2 | 3 | 4 | 5 |

**THANK YOU FOR YOUR HELP**

1. **ANNEX 3-PRE-PILOT INTERVIEW WITH TRAINEES (T0)**
2. **MIGRATION PATHWAY**
3. Can you tell me more about you? In particular, can you tell me which is your migration story e.g. where you come from and why did you leave your country?
4. **REPRESENTATION OF OLDER AGE**
5. What do you think “being old” means?
6. How are older people considered in your native country? (e.g., Which is their role in the community?)
7. Who takes care of older people in your native country?
8. **EXPECTATIONS ON HERO TRAINING AND PLAN FOR THE FUTURE**
9. Why did you choose to attend the HERO training?
10. What do you expect from the HERO training?
11. Have you got any fear or concern about the training?
12. Which are your plans for the next year? And for the next five years?
13. **PRE-PILOT FOCUS GROUP TOPIC-GUIDE WITH CARE STAFF (T0)**
14. Which are your expectations about the HERO program?
15. Which are the HERO strengths?
16. Which are the HERO weaknesses?
17. Which are the HERO opportunities?
18. Which are the HERO treats?
19. Have you got any concerns about the HERO training? If yes, which ones?
20. Have you got any concerns about the HERO internship? If yes, which ones?
21. How do you think to prevent the onset of these difficulties?
22. What do you think is needed such that the HERO program is successful and useful for trainees and care staff?
23. **NEX 7 MID-TERM INTERVIEW TO TRAINEES AND CARE STAFF (T1)**
24. Are you facing any difficulty during the training (for example with the subjects, with patients or in the relationships with trainers and colleagues)?
25. What would you like to change?
26. Which are the positive aspects of the training?
27. **ANNEX 2-POST-PILOT QUESTIONNAIRE FOR THE TRAINEES (T2)**

**THE WHOQOL-BREF**

**Instructions for interviewers**

This assessment asks how you feel about your quality of life, health, or other areas of your life. Please answer all the questions. If you are unsure about which response to give to a question, please choose the one that appears most appropriate. This can often be your first response.

Please keep in mind your standards, hopes, pleasures and concerns. We ask that you think about your life **in the last two weeks**. For example, thinking about the last two weeks, a question might ask:

|  |  | Not at all | Not much | Moderately | A great deal | Completely |
| --- | --- | --- | --- | --- | --- | --- |
|  | **Do you get the kind of support from others that you need?** | 1 | 2 | 3 | 4 | 5 |

I should circle the number that best fits how much support you got from others over the last two weeks. So you would circle the number 4 if you got a great deal of support from others. You would circle number 1 if you did not get any of the support that you needed from others in the last two weeks.

Please read each question, assess your feelings, and circle the number on the scale for each question that gives the best answer for you.

|  |  | Very poor | Poor | Neither poor  nor good | Good | Very good |
| --- | --- | --- | --- | --- | --- | --- |
| 1 | How would you rate your quality of life? | 1 | 2 | 3 | 4 | 5 |

|  |  | Very dissatisfied | Dissatisfied | Neither satisfied  nor dissatisfied | Satisfied | Very satisfied |
| --- | --- | --- | --- | --- | --- | --- |
| 2 | How satisfied are you with your health? | 1 | 2 | 3 | 4 | 5 |

The following questions ask about how much you have experienced certain things in the last two weeks.

|  |  | Not at all | A little | A moderate amount | Very much | An extreme amount |
| --- | --- | --- | --- | --- | --- | --- |
| 3 | To what extent do you feel that (physical) pain prevents you from doing what you need to do? | 1 | 2 | 3 | 4 | 5 |
| 4 | How much do you need any medical  treatment to function in your daily life? | 1 | 2 | 3 | 4 | 5 |
| 5 | How much do you enjoy life? | 1 | 2 | 3 | 4 | 5 |
| 6 | To what extent do you feel your life to be meaningful? | 1 | 2 | 3 | 4 | 5 |
| 7 | How well are you able to concentrate? | 1 | 2 | 3 | 4 | 5 |
| 8 | How safe do you feel in your daily life? | 1 | 2 | 3 | 4 | 5 |
| 9 | How healthy is your physical environment? | 1 | 2 | 3 | 4 | 5 |
| 10 | Do you have enough energy for everyday life? | 1 | 2 | 3 | 4 | 5 |
| 11 | Are you able to accept your bodily appearance? | 1 | 2 | 3 | 4 | 5 |
| 12 | Do you have enough money to meet your needs? | 1 | 2 | 3 | 4 | 5 |
| 13 | How available to you is the information that you need in your day-to-day life? | 1 | 2 | 3 | 4 | 5 |
| 14 | To what extent do you have the opportunity for leisure activities? | 1 | 2 | 3 | 4 | 5 |

|  |  | Very poor | Poor | Neither poor  nor good | Good | Very good |
| --- | --- | --- | --- | --- | --- | --- |
| 15 | How well are you able to get around? | 1 | 2 | 3 | 4 | 5 |

The following questions ask you to say how good or satisfied you have felt about various aspects of your life **over the last two weeks**.

|  |  | Very dissatisfied | Dissatisfied | Neither satisfied  nor dissatisfied | Satisfied | Very satisfied |
| --- | --- | --- | --- | --- | --- | --- |
| 16 | How satisfied are you with your sleep? | 1 | 2 | 3 | 4 | 5 |
| 17 | How satisfied are you with your ability to perform your daily living activities? | 1 | 2 | 3 | 4 | 5 |
| 18 | How satisfied are you with your capacity for work? | 1 | 2 | 3 | 4 | 5 |
| 19 | How satisfied are you with yourself? | 1 | 2 | 3 | 4 | 5 |
| 20 | How satisfied are you with your personal relationships? | 1 | 2 | 3 | 4 | 5 |
| 21 | How satisfied are you with your sex life? | 1 | 2 | 3 | 4 | 5 |
| 22 | How satisfied are you with the support you get from your friends? | 1 | 2 | 3 | 4 | 5 |
| 23 | How satisfied are you with the conditions of your living place? | 1 | 2 | 3 | 4 | 5 |
| 24 | How satisfied are you with your access to health services? | 1 | 2 | 3 | 4 | 5 |
| 25 | How satisfied are you with your transport? | 1 | 2 | 3 | 4 | 5 |

The following question refers to how often you have felt or experienced certain things in the last two weeks.

|  |  | Never | Seldom | Quite often | Very often | Always |
| --- | --- | --- | --- | --- | --- | --- |
| 26 | How often do you have negative feelings such as blue mood, despair, anxiety, depression? | 1 | 2 | 3 | 4 | 5 |

**THANK YOU FOR YOUR HELP**

1. **ANNEX 5-POST-PILOT INTERVIEW WITH TRAINEES**
2. **FEEDBACK ON THE TRAINING**
3. Can you tell me three strengths of the training?
4. Can you tell me three treats/weaknesses of the training?
5. What did you like more?
6. What did you dislike more?
7. Which were the main difficulties you had to face during the training?
8. In light of your answers above, what would you like to change in the training?

1. **REPRESENTATION OF OLDER AGE**
2. What do you think “being old” means?
3. In your opinion, how are older people considered in [please enter the appropriate *HERO study country*]?
4. What it was like to take care of a sick older person?
5. Can you tell me the positive and negative aspects of elderly healthcare?

1. **RELATIONAL EXPERIENCE DURING THE TRAINING**
2. How did you feel during the HERO training (happy, sad, frustrated, relaxed, angry, tired…)?
3. Can you define the relationship with the other trainees?
4. Can you tell me more about your relationship with the hospital/facility staff, namely with manager and nurses?
5. Can you describe your relationship with older care recipients? Can you tell me any episode you remember that can help me understand your experience with the patients?

1. **PLAN FOR THE FUTURE**
2. Which are your plans for the next year? And for the next five years?
3. Where would you like to work in the future?
4. **ANNEX 6-POST-PILOT FOCUS-GROUP TOPIC-GUIDE WITH CARE STAFF**

**Methodological note:** The focus-group will be led by a researcher in the role of moderator with the support of another researcher who will take notes, control the time and observe the communication dynamics within the group. The conversation will be recorded and the contents will be transcribed verbatim in the national language. Should the interviewees not give their consent, the answers will be written by the interviewer and the note taker. Then the contents will be analysed thematically [1-3] by the national teams with the support and supervision of INRCA researchers. Just some quotations will be translated from national languages into English to be included in the study findings report and in research articles.

1. **FEEDBACK ON TRAINING CONTENTS AND ORGANISATION**
2. What do you think about the training contents (i.e. the curriculum)? Did it provide the appropriate knowledge, skills and competences to the trainees? Is there anything missing and to add?
3. What do you think have been the main strengths of the training?
4. Which were the main weaknesses?
5. Which were the opportunities?
6. Which were the treats?
7. What resources did you put in place for overcoming the difficulties that arose during the HERO program?
8. **FEEDBACK ON THE TRAINEES**
9. Which were the main difficulties you faced with the trainees during the “on the job” training?
10. What about the trainees’ recognition of the hospital/facility vision, mission and organisation?
11. What was the trainees’ attitude and behavior like during the training?
12. Are there any cultural aspects you want to underline that represent a bias or a strength for the relationship between the learners and the older patients?
13. How can this organization prepare a career pathway for the learners?
14. **SUGGESTIONS TO HEALTHCARE MANAGERS, EDUCATIONAL ORGANISATIONS AND POLICY MAKERS**
15. Which is the “lesson learned” by the HERO experience?
16. Which is the suggestion you would like to give to the National/Regional health managers and policy makers about the training and the employment of migrants and refugees in the elderly healthcare sector in the Country?
17. How the training of migrants and refugees can be funded and replicated in the future?
